# Advances in physiotherapy for chronic low-back pain: a level I systematic review

**DOI:** 10.1186/s10195-026-00972-0

**Published:** 2026-10-18

**Authors:** Filippo Migliorini, Nicola Maffuli, Luise Schäfer, Nicola Manocchio, Michela Bossa, Calogero Foti

**Affiliations:** 1 Department of Trauma and Reconstructive Surgery, University Hospital of Halle, Martin-Luther University Halle-Wittenberg, Ernst-Grube-Street 40, 06097 Halle, Saale Germany; 2 Department of Orthopaedic and Trauma Surgery, Academic Hospital of Bolzano (SABES-ASDAA), 39100 Bolzano, Italy; 3 Department of Life Sciences, Health, and Health Professions, Link Campus University, 00165 Rome, Italy; 4 Department of Trauma and Orthopaedic Surgery, Faculty of Medicine and Psychology, University “La Sapienza” of Rome, Rome, Italy; 5 School of Pharmacy and Bioengineering, Keele University Faculty of Medicine, Stoke on Trent, ST4 7QB UK; 6 Centre for Sports and Exercise Medicine, Barts and the London School of Medicine and Dentistry, Mile End Hospital, Queen Mary University of London, London, E1 4DG UK; 7 Clinical Sciences and Translational Medicine Department, Tor Vergata University, Rome, Italy; 8 Behavioral Neuropsychology Laboratory, I.R.C.C.S. “Santa Lucia” Foundation, Rome, Italy

**Keywords:** Rehabilitation, Biomechanics, Neuromuscular, Kinesiophobia, Proprioception, Telerehabilitation, Biopsychosocial

## Abstract

**Background:**

Non-pharmacologic management of chronic low back pain (cLBP) should be considered the first-line treatment. However, consensus guidelines and internationally accepted recommendations on the therapeutic algorithm are missing. The present level I systematic review presents a qualitative analysis of advancements in physiotherapy over the past decades to discuss the role of physical and rehabilitation medicine in the management of cLBP.

**Methods:**

In August 2025, the following databases were accessed: PubMed, Web of Science and Embase. All randomised controlled trials evaluating the efficacy of physiotherapy programs in patients with cLBP were assessed using the Preferred Reporting Items for Systematic Reviews and Meta-Analyses (PRISMA) criteria. The data extraction and risk-of-bias evaluation were performed independently by two authors.

**Results:**

Data from 150 randomised controlled trials (RCTs) (12,773 patients) were collected. The mean symptom duration was 61.2 ± 51.0 months, and the mean follow-up was 4.3 ± 5.9 months. The mean age was 44.5 ± 9.4 years, and the mean body mass index (BMI) was 25.8 ± 2.9 kg/m2.

**Conclusions:**

Managing cLBP is challenging. While individual investigations highlight the efficacy of a specific intervention, most cLBP management strategies share common elements. Combining key elements from various approaches may prove crucial. Although this result is not decisive for treatment, it paves the way for further research that considers a more holistic and multidisciplinary approach.

**Supplementary Information:**

The online version contains supplementary material available at https://doi.org/10.1186/s10195-026-00972-0.

## Introduction

Low back pain (LBP) is defined as pain, muscle tension or stiffness localised in the lumbar (L1–L5 vertebral region) and sacral spine (S1 to sacrococcygeal junction region) [1]. This condition can be accompanied by radicular LBP or radicular leg pain, secondary to nerve root impingement or dorsal root ganglion irritation, which presents as lower extremity pain, paresthesia and leg(s) weakness along a dermatomal distribution [2].

Chronic low back pain (cLBP), defined as LBP lasting more than 3 months, is a globally prevalent health problem with a high medical and economic burden on both individuals and society [3, 4]. People suffering from cLBP do indeed show prolonged disability, leading to poorer physical function, more restrictions and depressive symptoms and lower health-related quality of life (QoL), but also to a decline in productivity [3, 5].

In cLBP, various interventions are effective, such as pharmacological and non-pharmacological treatments, including physical and rehabilitation treatments, psychological treatments and complementary and alternative medicine approaches [6–8]. In particular, recent guidelines strongly recommend initially selecting non-pharmacologic treatments, including therapeutic exercise, educational techniques, massage techniques, postural therapies, physical agents, complementary medicine techniques and more [9–11].

Re-educational programs have become essential to managing cLBP and are often used with pharmacological and surgical interventions [12, 13]. This review presents a qualitative analysis of the advances in physiotherapy over the past decades to highlight the role of physical and rehabilitation medicine in cLBP treatment.

## Methods

### Eligibility criteria

All the randomised controlled trials (RCTs) evaluating the efficacy of physiotherapy programs in patients with cLBP were accessed. According to the authors’ language capabilities, English, German, Italian, French and Spanish articles were eligible. According to the Oxford Centre of Evidence-Based Medicine [14], only RCTs with level I of evidence were considered. Reviews, opinions, letters and editorials were not considered. Animals, in vitro, biomechanics, computational and cadaveric studies were not eligible. Studies reporting on non-specific [15] or mechanical [16] cLBP were included. The pain was defined as chronic when symptoms persisted for at least 3 months [7]. Studies including patients with radiculopathy and neurologic symptoms were excluded from this analysis.

### Search strategy

This study was conducted according to the Preferred Reporting Items for Systematic Reviews and Meta-Analyses (PRISMA) 2020 statement [17]. The following algorithm was established:

- Problem: cLBP;
- Intervention: physiotherapy;
- Comparison: different modalities of physiotherapy;
- Design: RCT.

In August 2025, the following databases were accessed: PubMed, Web of Science and Embase. No time constraint was set for the search, which was restricted to only RCTs. The medical subject headings (MeSH) used in PubMed are in the appendix. No additional filters were used in the database search.

### Selection and data collection

Two authors (M.N., L.S.) performed the database search. Disagreements were settled by a third author (N.M.) with long experience in systematic reviews. All the resulting titles were screened by hand, and the abstract was accessed if suitable. If the abstract matched the topic, the full text was accessed. If the full text was unavailable, the article was not considered for inclusion. A cross-reference of the full-text bibliography was also conducted to identify additional studies. All PDFs of full texts were saved in a dedicated folder and shared between the authors in a private cloud. Duplicates were deleted. Study selection and collection lasted 3 months, and the search was updated at each revision phase.

### Data items

Two authors (M.N. and L.S.) independently performed data extraction. The following data at baseline were extracted: author and year of publication, journal of publication, men:women ratio, number of patients, included with related mean age and body mass index (BMI; kg/m^2^), mean length of symptoms duration before physiotherapy and the length of follow-up. Data were extracted in Microsoft Office Excel version 16.72 (Microsoft Corporation, Redmond, USA).

### Assessment of the risk of bias and quality of the recommendations

The risk of bias was evaluated in accordance with the guidelines in the Cochrane Handbook for Systematic Reviews of Interventions [18]. Two reviewers (M.N. and L.S.) independently assessed the risk of bias in the extracted studies. Disagreements were solved by a third senior author (N.M.). RCTs were evaluated using the risk of bias of the software Review Manager 5.3 (The Nordic Cochrane Collaboration, Copenhagen). The following endpoints were evaluated: selection, detection, performance, attrition, reporting and other biases.

### Synthesis methods

The principal author (F.M.) performed the statistical analyses following the recommendations of the Cochrane Handbook for Systematic Reviews of Interventions [19]. For descriptive statistics, IBM SPSS version 25 was used. The mean and standard deviation were used. To assess baseline comparability, data distribution was analysed using the Shapiro–Wilk test. Analysis of variance (ANOVA) and the Kruskal–Wallis test were used for parametric and non-parametric data, with *P* values > 0.1 considered satisfactory. Given the substantial heterogeneity in intervention protocols, comparator groups, follow-up durations, outcome measures and reporting formats, no formal quantitative synthesis was attempted, and the findings were therefore interpreted qualitatively.

## Results

### Study selection

**Fig. 1:**
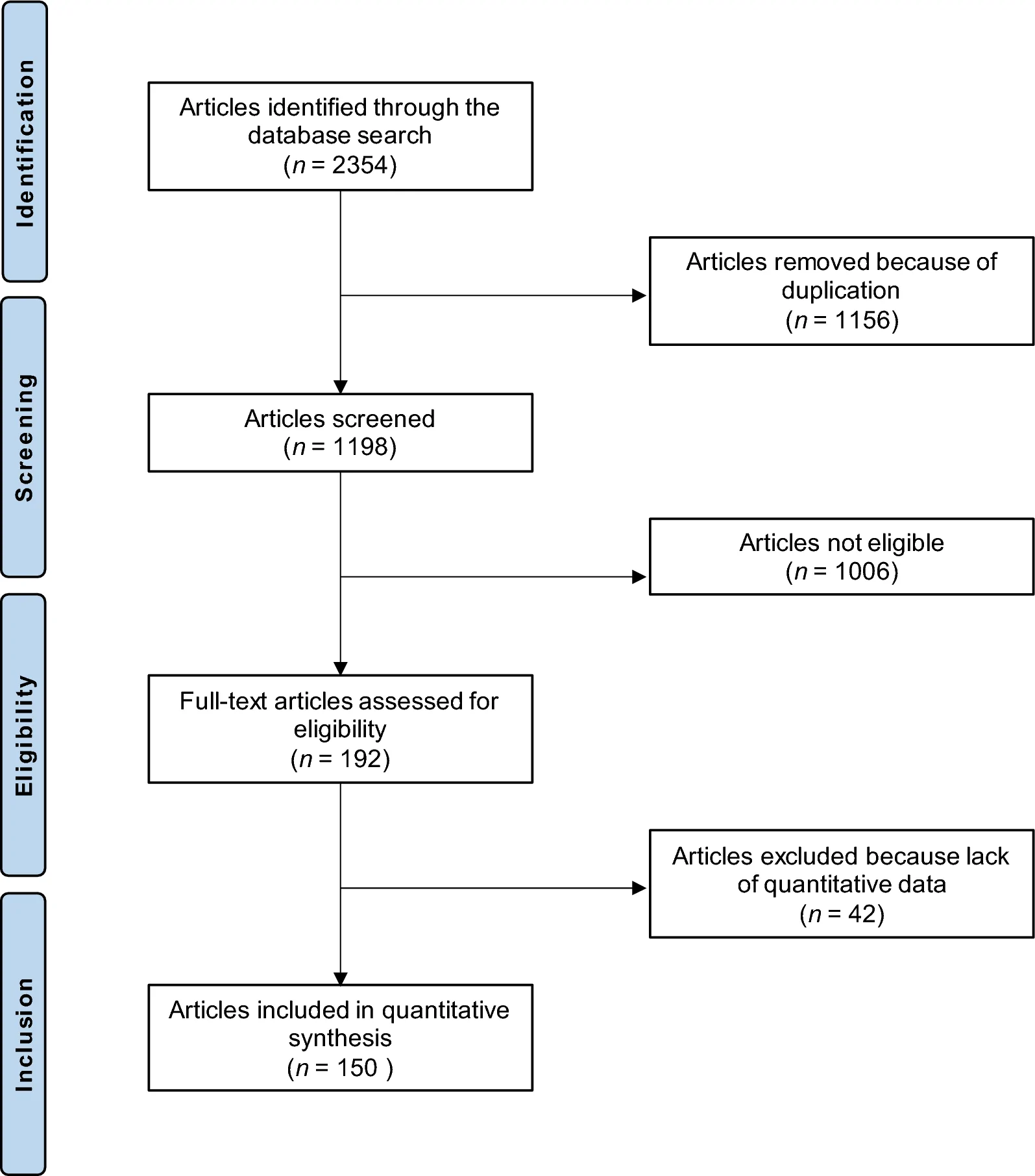
PRISMA flow chart of the literature search

After a total of 2354 RCTs were retrieved, 1156 studies were excluded because they were duplicates. Another 1006 articles did not fulfil the eligibility criteria and were therefore discarded. Reasons for non-inclusion include, in detail: study design (*N* = 697), low level of evidence (*N* = 148), therapy protocols that could not be classified into one of the categories of interest (*N* = 149) and language limitations (*N* = 12). After full-text evaluation, an additional 42 investigations were excluded because quantitative data on the outcomes of interest were not available. Finally, 150 RCTs were available for inclusion. The results of the literature search are shown in Fig. 1.

### Risk of bias assessment

**Fig. 2:**
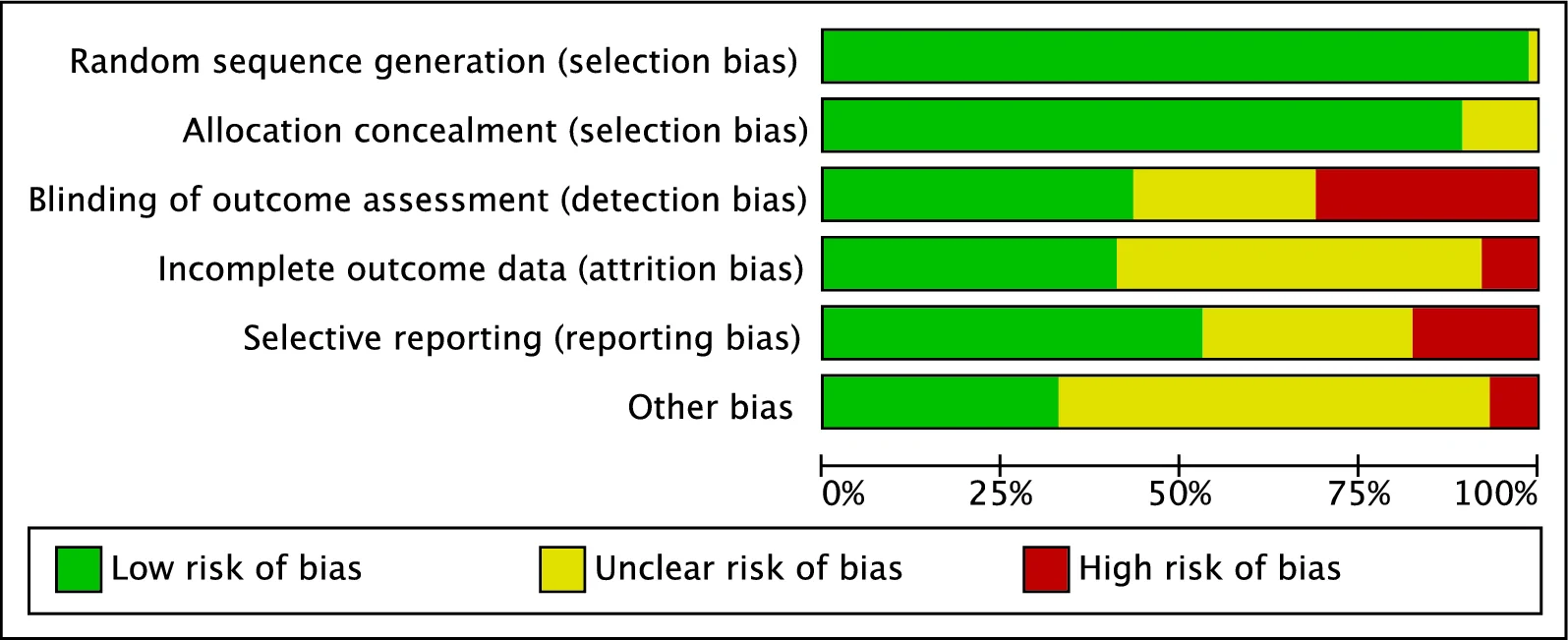
Cochrane risk of bias tool

The risk of bias analysis showed a low risk of selection bias, as all included studies were RCTs. The allocation of patients to each treatment group was performed with a high degree of quality in most studies, resulting in a low to moderate risk of allocation bias. Moderate risk was present for detection and performance bias, attributed to the lack of information on the blinding of investigators and patients during treatment and follow-up. In some studies, information on study dropouts during enrolment or analysis was incompletely reported, resulting in moderate attrition bias. The risk of reporting bias was overwhelmingly moderate, and the risk of other biases was primarily low. In summary, the risk of bias graph indicates a moderate quality methodological assessment of RCTs (Fig. 2).

### Study characteristics and results of individual studies

Data from 12,773 patients were collected. The mean symptom duration was 61.2 ± 51.0 months, and the mean follow-up was 4.3 ± 5.9 months. The mean age was 44.5 ± 9.4 years. The mean BMI was 25.8 ± 2.9 kg/m^2^. The generalities and demographics of the included studies are presented in Table 1.

**Table 1:** Generalities and patient baseline of the included studies

Author, year	Journal	Class of treatment	Type of movement	Type of treatment	Patients (*n*)	Mean age	Women (%)
Aasa et al. 2015 [84]	*J Orthop Sports Phys Ther*	Exercise	Low-load	Low-load motor control exercise	25	42.0	54
		Exercise	High-load lifting	High-load lifting exercise	28	42.0	57
Balthazard et al. 2012 [116]	*BMC Musculoskelet Disord*	Spinal manipulation	High velocity, low amplitude	Spinal manipulation and active exercise (mobility, passive stretching, motor control and strengthening)	19	44.0	36
		Physical agents	Ultrasound	Detuned US and active exercise (mobility, passive stretching, motor control and strengthening)	18	42.0	30
Bhadauria et al. 2017 [94]	*J Exerc Rehabil*	Exercise	Stabilisation	Stabilisation with verbal cues and tactile facilitation	12	32.8	50
		Exercise	Strengthening	Dynamic strengthening	12	36.7	42
		Pilates	Contraction	Isometric contraction	12	35.3	8
Cecchi et al. 2010 [119]	*Clin Rehabil*	Back school	Individualised	Back school	68	57.9	70
		Physiotherapy	Individualised	Mobilisation, active exercise, massage treatment of the soft tissues, proprioceptive neuromuscular facilitation	68	60.5	61
		Spinal manipulation	Mobilisation, manipulation	Spinal manipulation	69	58.1	69
Costa et al. 2009 [102]	*Phys Ther*	Motor control exercise	Individualised	Motor control exercise	77	54.6	58
		Sham		Detuned US and detuned short-wave therapy	77	52.8	62
Vibe Fersum et al. 2019 [157]	*Eur J Pain*	Spinal manipulation	Individualised	Joint mobilisation or manipulation (spine and pelvis)	59	43.1	52
		Cognitive functional therapy		Cognitive-behavioural	62	42.9	53
Garcia et al. 2013 [136]	*Phys Ther*	Back school	Unknown	Back school	74	54.2	69
		Mckenzie	Symptom guided	Educational component and postural training (Mckenzie)	74	53.7	78
Goldby et al. 2006 [88]	*Spine*	Spinal Stabilisation	Stabilisation	Spinal Stabilisation and back school	35	43.4	68
		Spinal manipulation	Individualised	Spinal manipulation and back school	37	41.0	70
		Control		Back school	19	41.5	68
Halliday et al. 2016 [134]	*J Orthop Sports Phys Ther*	Mckenzie	Symptom guided	Postural training (Mckenzie)	32	48.8	80
		Motor control exercise	Contraction	Motor control exercise	30	48.3	80
Hohmann et al. 2018 [50]	*Dtsch Arztebl Int*	Hirudotherapy		Hirudotherapy and back school	25	59.3	88
		Exercise	Various	Exercise and back school	19	56.5	95
Kääpä et al. 2006 [187]	*Spine*	Multidisciplinary	Various	Group multidisciplinary rehabilitation: cognitive-behavioural stress management and applied relaxation sessions, back school education including occupational intervention and physical exercise program	59	46.0	98
		Physiotherapy	Various	Individual physiotherapy: light active exercise (muscle stretching, spine mobilisation and deep trunk muscle exercises) and passive treatment (massage, spine traction, spinal mobilisation and TENS)	61	46.5	
Kobayashi et al. 2019 [172]	*Complement Ther Med*	Shiatsu	Various	Shiatsu and standard care (compress or oral medicine)	30	67.4	67
		Standard care		Standard care (compress or oral medicine)	29	68.3	62
Lawand et al. 2015 [130]	*Joint Bone Spine*	Exercise	Stretching	Global postural reeducation (Souchard)	30	49.4	81
		Control		Drugs	30	47.5	73
Macedo et al. 2019 [164]	*Physiotherapy*	Kinesio taping with tension	Traction	Kinesiotaping	27	25.0	100
		Kinesio taping no tension		Kinesiotaping	27	24.0	
		Sham		Sham tape	27	25.0	
		Control			27	24.0	
Majchrzycki et al. 2014 [127]	*Sci World J*	Massage	Deep tissue massage	Deep tissue massage	28	52.6	46
		Massage	Deep tissue massage	Deep tissue massage and NSAID	26	50.8	50
Murtezani et al. 2015 [137]	*J Back Musculoskelet Rehabil*	Mckenzie	Symptom guided	Mckenzie	110	48.8	25
		Physical agents	Various	Interferential current, US and heat	109	47.5	62
Sahin et al. 2018 [150]	*Turk J Phys Med Rehab*	Physical agents	Various	PT (hot pack,US and TENS treatment) and exercise (strengthening and stretching)	50	50.4	64
		Control	Stretching and strengthening	Active isotonic and isometric strengthening exercises, stretching exercises	50	46.2	62
Saper et al. 2017 [169]	*Ann Intern Med*	Yoga	Various	Yoga	127	46.4	57
		Aerobics	Various	Exercise	129	46.4	70
		Education		Education	64	44.2	66
Suh et al. 2019 [98]	*Med*	Stretching	Stretching	Stretching	13	53.5	62
		Walking exercise	Walking	Walking exercise (WE)	13	54.2	85
		Spinal stabilisation	Stabilisation	Spinal stabilisation	10	57.4	60
		Spinal stabilisation	Stabilisation, walking	Stabilisation and WE	12	54.8	67
Takahashi et al. 2017 [76]	*Fukushima J Med Sci*	Control		NSAIDs	15	53.3	53
		Exercise	Stretching and strengthening	Strengthening and stretching	18	57.6	56
Uzunkulaoğlu et al. 2018 [165]	*Turk J Phys Med Rehabil*	Kinesio taping with tension	Traction	Kinesiotaping with tension	30	21.6	63
		Kinesio taping without tension		Kinesiotaping without tension	30	21.3	63
Yeung et al. 2003 [154]	*J Altern Complement Med*	Exercise	Various	Warm up and stretching exercises	26	55.6	81
		Exercise	Various	Exercises and electroacupunture	26	50.4	85
Dufour et al. 2010 [183]	*Spine*	Exercise	Strengthening	Aerobic training and strengthening exercises and education	129	41.2	57
		Exercise	Strengthening	Strengthening exercises (intensive muscle training)	143	40.6	56
Helmhout et al. 2004 [85]	*Spine*	Exercise	High-intensity strengthening	Progressive resistance muscle training	41	41.0	0
		Exercise	Low-intensity strengthening	Non-progressive, low-intensity resistance training	40	40.0	
Jarzem et al. 2005 [144]	*J Musculoskelet Pain*	Sham		Sham	83	45.1	50
		TENS		TENS	84		
		Acupuncture TENS		Acupuncture TENS	78		
		TENS	Biphasic	TENS	79		
Meng et al. 2011 [51]	*Clin J Pain*	Back school		Back school	181	50.2	65
		Back school		Back school	163	49.5	63
Prommanon et al. 2015 [155]	*J Phys Ther Sci*	Back care pillow		US, hot pack and back care pillow	26	38.5	42
		Control		US and hot pack	26	39.7	50
Tavafian et al. 2011 [189]	*Clin J Pain*	Education		Education and drugs	97	44.6	73
		Control		Control and drugs	100	45.9	83
Tavafian et al. 2014 [190]	*Int J Rheum Dis*	Education		Education and drugs	87	44.6	75
		Control		Control and drugs	91	46.2	82
Alfuth et al. 2016 [34]	*Orthopäde*	Mobilisation	Mobilisation	Mobilisation	14	50.0	79
		Stabilisation	Stabilisation	Stabilisation	13	43.0	54
Grande-Alonso et al. 2019 [185]	*Pain Med*	Multidisciplinary	Various	Multidisciplinary	25	39.9	56
		Multidisciplinary	Stabilisation	Multidisciplinary	25	38.3	56
Ali et al. 2019 [120]	*J Bodyw Mov Ther*	Spinal manipulation	Various	Spinal manipulation	14	35.4	
		Spinal manipulation	Various	Spinal manipulation	14	35.3	
Ahmadi et al. 2020 [79]	*Clin Rehabil*	Exercise	Individualised	Exercise (Feldenkrais method) and education	30	42.6	100
		Education	Individualised	Home-based exercise and education	29	38.9	100
Almhdawi et al. 2020 [192]	*Clin Rehabil*	Exercise	Strengthening, stretching	Strengthening and stretching exercises	21	40.5	34
		Control			20	41.7	20
Added et al. 2016 [163]	*J. Orthop. Sports Phys. Ther*	Physiotherapy	Individualised	Manual therapy exercises	74	44.6	72
		Physiotherapy	Individualised	Exercises and Kinesio taping	74	45.6	72
Arampatzis et al. 2017 [105]	*Eur J Appl Physiol*	Exercise	Low–moderate intensity	Low–moderate intensity exercises	20	31.9	40
		Control			20	31.4	45
Areeudomwong et al. 2016 [97]	*Musculoskeletal Care*	Exercise	Contraction	Proprioceptive neuromuscular facilitation and contraction exercises	21	35.4	71
		Control			21	36.2	76
Bae et al. 2018 [59]	*J Back Musculoskelet Rehabil*	Exercise	Core stabilisation	Core stabilisation	18	32.7	50
		Exercise	Strengthening	Strengthening exercises	18	32.4	61
Bi et al. 2013 [75]	*Int J Med Res*	Exercise	Contraction	Pelvic floor muscle exercise	23	29.1	44
		Control	Strengthening	US, short-wave diathermy and strengthening exercises	24	30.9	46
Bicalho et al. 2010 [117]	*Man Ther*	Manipulation	High-velocity	Lumbar manipulation	20	29.5	75
		Control			20	26.5	60
Bronfort et al. 2011 [80]	*Spine J*	Exercise	Various	Education and simple exercises	101	45.6	58
		Spinal manipulation	High-velocity, low-amplitude	Spinal manipulation	100	45.2	66
		Exercise	Strengthening	Strengthening exercises	100	44.5	57
Cai et al. 2017 [101]	*Med Sci Sports Exerc*	Exercise	Resistance	Resistance exercises	25	28.9	50
		Exercise	Contraction	Isometric contraction	24	26.1	
		Exercise	Stabilisation	Stabilisation	25	26.9	
Azevedo et al. 2017 [52]	*Phys Ther*	Exercise	Strengthening, stretching	Strengthening and stretching exercises	74	40.4	58
		Control	Various	Education and exercises	74	43.4	65
Castro-Sánchez et al. 2016 [118]	*Spine J*	Spinal manipulation	High-velocity	Manipulation	31	43.0	65
		Spinal manipulation	Low-velocity	Manipulation	31	47.0	61
Ryan et al. 2010 [42]	*Man Ther*	Exercise	Various	Aerobic exercises	20	45.2	70
		Control		Education	18	45.5	61
Chhabra et al. 2018 [78]	*Eur Spine J*	App	Various	Exercises	45	41.4	
		Control		Drugs	48	41.0	
Cortell-Tormo et al. 2018 [95]	*J Back Musculoskelet Rehabil*	Exercise	Various	Exercises for coordinated contraction of transversus abdominis with lumbar multifidus	11	35.6	100
		Control		No intervention	8	35.6	100
Cruz-Díaz et al. 2015 [138]	*Disabil Rehabil*	Pilates	Individualised	Strengthening exercises	53	69.6	100
		Physiotherapy	Various	TENS	48	72.7	100
Cruz-Díaz et al. 2017 [193]	*Complement Ther Med*	Pilates	Various	Pilates	34	36.9	68
		Pilates	Various	Pilates	34	35.5	62
		Control		No intervention	30	36.3	63
Cuesta-Vargas et al. 2011 [182]	*Am J Phys Med Rehabil*	Multidisciplinary	Various	Multimodal rehab	24	37.6	58
		Multidisciplinary	Various	Multimodal rehab	25	39.8	54
Diab et al. 2013 [99]	*J Back Musculoskelet Rehabil*	Exercise	Traction	Lumbar traction	40	46.3	45
		Exercise	Stretching	Strengthening exercises	40	45.9	43
Koldaş Doğan et al. 2008 [142]	*Clin Rheumatol*	Aerobics	Walking	Aerobic exercises	19	37.1	79
		Physical agents	Various	Hot packs and US and TENS	18	41.5	78
		Control		Mobilisation and stretching	18	42.1	78
Eardley et al. 2013 [184]	*Forsch Komplementmed*	Multidisciplinary	Individualised	Therapeutic conversation	20	48.8	85
		Sham	Individualised	Sham exercises	21	48.1	67
		Delayed			17	44.6	65
Engbert et al. 2011 [161]	*Spine*	Exercise	Climbing	Climbing exercises	10	51.9	60
		Exercise	Various	Trunk stabilisation and strengthening	13	50.4	46
de Oliveira et al. 2013 [194]	*Phys Ther*	Spinal manipulation	High-velocity	Spinal manipulation	74	46.0	68
		Spinal manipulation	Region-specific	Spinal manipulation	74	46.3	80
França et al. 2012 [96]	*J Manipulative Physiol Ther*	Exercise	Stabilisation	Spinal stabilising exercises	15	42.1	
		Exercise	Stretching	Strengthening exercises	15	41.5	
Friedrich et al. 1998 [70]	*Arch Phys Med Rehabil*	Exercise	Various	Motivational intervention	44	43.3	57
		Exercise	Various	Spinal mobility and trunk and lower limbs exercises	49	44.9	45
Frost et al. 1995 [81]	*BMJ*	Exercise	Aerobics	Stretching and aerobic exercises	36	34.2	53
		Control		Back school	35	38.5	51
Garcia et al. 2017 [131]	*BMJ*	Mckenzie	Various	Mckenzie	74	57.5	78
		Control		Detuned pulsed ultrasound	73	55.5	74
Gardner et al. 2019 [71]	*BMJ*	Exercise	Individualised	Individualised exercises	37	44.0	66
		Control	Various	Exercise	38	45.0	49
Gavish et al. 2015 [68]	*Physiotherapy*	Exercise	Oscillation	Mobilisation	18	53.2	33
		Control		No intervention	18	47.1	56
Geisser et al. 2005 [123]	*Clin J Pain*	Exercise	Various	Self-corrections and stretches and strengthening exercises	21	39.3	67
		Exercise	Various	Exercises and sham manipulation	18	38.7	56
		Non-specific exercise	Various	Non-specific exercise	15	36.5	80
		Non-specific exercise	Various	Non-specific exercise and sham manipulation	18	46.3	61
Gwon et al. 2020 [149]	*Physiother Theory Pract*	Exercise	Side bridge	Vibration and side-lying bridge exercise on a sling suspension system	15	21.9	2
		Exercise	Side bridge	Side-lying bridge exercise on a sling suspension system	15	21.6	2
Haas et al. 2014 [121]	*Spine J*	Sham	Various	Sham manipulation	95	40.9	49
		Spinal manipulation	High velocity, low amplitude	Spinal manipulation	99	41.4	49
		Spinal manipulation	High velocity, low amplitude	Spinal manipulation	97	41.8	49
		Spinal manipulation	High velocity, low amplitude	Spinal manipulation	100	41.2	52
Halliday et al. 2019 [135]	*Physiotherapy*	Mckenzie	Symptom guided	Mckenzie	35	48.8	80
		Motor control exercise	Contraction	Motor control exercises	35	48.3	80
Harts et al. 2008 [195]	*Aust J Physiother*	Exercise	High intensity	High-intensity progressive resistance exercise	23	44.0	0
		Exercise	Low intensity	Low-intensity resistance exercise program	21	42.0	
		Control		No intervention	21	41.0	
Macedo et al. 2015 [110]	*Phys Ther*	Exercise	Various	Individualised and submaximal exercises	86	49.6	52
		Motor control exercise	Symptom guided	Spine and pelvis stabilisation	86	48.7	66
Javadian et al. 2012 [60]	*J Back Musculoskelet Rehabil*	Exercise	Stabilisation	Spinal stabilisation	30		
		Exercise	Various	Active mobilisation exercises			
Loss et al. 2020 [196]	*Chiropr Man Ther*	Spinal manipulation	Thrust	Lumbar manipulation	12	41.7	50
		Control	Various	Sham manipulation	12	43.9	50
Kell et al. 2011 [197]	*J Strength Cond Res*	Exercise	Strengthening	Strengthening exercises	60	42.4	31
		Exercise	Strengthening	Strengthening exercises	60	41.7	37
		Exercise	Strengthening	Strengthening exercises	60	42.8	33
		Control		No intervention	60	43.2	38
Kim et al. 2015 [141]	*Clin Rehabil*	Exercise	Contraction	Core exercises and TENS	27	29.7	100
		Control		TENS	26	28.6	
Kim et al. 2018 [198]	*J Sport Rehabil*	Exercise	Various	Exercises using a sling	38	39.5	61
		Exercise	Stabilisation	Stabilizing exercise	39	46.2	54
Tekur et al. 2008 [167]	*J Altern Complement Med*	Yoga	Various	Yoga	40	49.0	53
		Exercise	Various	Active mobilisation exercises	40	48.0	38
Tekur et al. 2012 [166]	*Complement Ther Med*	Yoga	Various	Yoga	40	49.0	53
		Exercise	Various	Active mobilisation exercises	40	48.0	38
de Oliveira et al. 2020 [199]	*J Physiother*	Spinal manipulation	High-velocity	High-velocity thrust manipulation	71	45.0	77
		Spinal manipulation	Various	‘Generic manipulation’	72	45.0	78
Zou et al. 2019 [173]	*Medicina*	Tai chi	Various	Tai chi	15	58.1	73
		Exercise	Stabilisation	Bridge exercises	15	58.4	73
		Control		No intervention	13	60.7	77
Zhang et al. 2014 [38]	*J Int Med Res*	Education	Strengthening	Education sessions once a week for 12 weeks	25	22.3	33
		Control	Strengthening	Lumbar strengthening exercises	24	23.0	41
Zheng et al. 2012 [124]	*J Tradit Chin Med*	Massage	Pressure; traction	Deep massage to the tender point and peripheral taut band	30	43.0	44
		Control	Traction	Sham manipulation	30	42.0	50
Yang et al. 2021 [180]	*J Bodyw Mov Ther*	Pilates	Various	Pilates	20	50.5	75
		Control		Education program regarding low back pain	19	47.9	79
Waseem et al. 2018 [200]	*J Back Musculoskelet Rehabil*	Exercise	Core Stabilisation	Core and pelvic floor exercises	53	46.4	34
		Exercise	Various	Stretching	55	45.5	35
Williams et al. 2005 [171]	*Pain*	Yoga	Various	Yoga	20	48.7	65
		Control		Two 1-h lectures on occupationalphysical therapy	24	48.0	71
Verbrugghe et al. 2021 [86]	*Int J Environ Res Public Health*	Exercise	High-intensity strengthening	Cardiorespiratory training, general resistance training and core muscle training—high intensity	16	44.3	68
		Control	Moderate-intensity strengthening	Cardiorespiratory training, general resistance training and core muscle training—moderate intensity	13	44.0	68
Sipaviciene et al. 2020 [90]	*Clin Biomech*	Exercise	Stabilisation	Dynamic stretching exercises and lumbar stabilisation	35	38.3	100
		Exercise	Strengthening	Lumbar muscle strengthening exercise	35	38.5	100
Phattharasupharerk et al. 2018 [175]	*J Bodyw Mov Ther*	Qi gong	Various	Qi gong	36	35.7	67
		Control		General advice on managing low back pain	36	34.8	61
Magalhães et al. 2018 [109]	*Braz J Phys Ther*	Exercise	Various	Stretching exercises of main muscle groups and motor control exercises	33	46.6	76
		Exercise	Various	Progressive and sub-maximal exercises	33	47.2	73
Monticone et al. 2013 [158]	*Clin J Pain*	Exercise + Cognitive Behavioural Therapy	Various	Cognitive-behavioural	45	49.0	60
		Control	Various	Active and passive mobilisations of the spine, and exercises aimed at stretching and strengthening muscles, and improving postural control	45	49.7	56
Monticone et al. 2014 [107]	*Eur Spine J*	Motor control exercise + cognitive behavioural therapy	Stabilizing	Spinal stabilising exercises	10	58.9	70
		Control	Various	Passive spinal mobilisation, stretching, muscle strengthening and postural control	10	56.6	40
Morone et al. 2011 [43]	*Eur J Phys Rehabil Med*	Back school	Various	Theoretical lessons	41	61.2	59
		Control		Drugs	29	58.6	72
Matarán-Peñarrocha et al. 2020 [83]	*Clin Rehabil*	Exercise	Various	Supervised exercise programme	32	54.3	53
		Exercise		Non-supervised home exercise programme	32	53.2	47
Laosee et al. 2020 [129]	*Complement Ther Med*	Massage	Pressure	Traditional Thai massage	70	68.2	77
		Massage	Pressure	Traditional Thai massage	70	69.1	71
Rittweger et al. 2002 [148]	*Spine*	Exercise	Extension	Isodynamic lumbar extension exercise	25	49.8	44
		Exercise	Vibration	Whole body vibration	25	54.1	52
Prado et al. 2019 [112]	*Physiother Theory Pract*	Exercise	Stretching	Active exercises	27	35.0	70
		Control		No intervention	27	33.0	63
Vollenbroek-Hutten et al. 2004 [191]	*Clin Rehabil*	Multidisciplinary	Various	Multimodal rehab	69	38.5	
		Control		No intervention	73	39.5	
del Pozo-Cruz et al. 2011 [147]	*J Rehabil Med*	Exercise	Vibration	Whole body vibration	25	58.7	74
		Control		No intervention	24	59.5	72
Kostadinovic et al. 2020 [91]	*J Back Musculoskelet Rehabil*	Exercise	Stabilisation; mobilisation	Lumbar stabilisation exercises and thoracic mobilisation and TENS	40	44.1	55
		Exercise	Stabilisation	Lumbar stabilisation and TENS	40	44.3	58
Monticone et al. 2015 [106]	*Eur J Pain*	Exercise + cognitive behavioural therapy	Individualised	Exercises	75	53.2	63
		Control		Ergonomic advice	75	53.8	60
Járomi et al. 2018 [47]	*J Clin Nurs*	Back school	Various	Isometric and isotonic exercises	67	41.7	94
		Control		Brief written lifestyle guidance	70	41.1	93
Liu et al. 2019 [174]	*Int J Environ Res Public Health*	Tai chi	Various	Tai chi	15	58.1	73
		Exercise	Stabilisation	Active exercises (bridge)	15	58.4	73
		Control		No intervention	13	60.7	77
Lara-Palomo et al. 2012 [201]	*Clin Rehabil*	Massage	Interferential current	Interferential current	30	50.0	70
		Massage	Superficial pressure	Superficial manual massage	31	47.0	65
Saha et al. 2019 [128]	*Complement Ther Clin Pract*	Massage	Pressure	Spinal manipulation	25	52.2	68
		Control		No intervention	25	47.2	88
Segal-Snir et al. 2016 [72]	*J Back Musculoskelet Rehabil*	Exercise	Rotation	Rotation exercises	20	57.2	100
		Control		No intervention	15	54.7	100
Nambi et al. 2014 [168]	*Int J Yoga*	Yoga	Various	Yoga	30	44.3	63
		Control	Strengthening, stretching	Strengthening and stretching of the abdominal and back muscles	30	43.7	43
Salamat et al. 2017 [108]	*J Bodyw Mov Ther*	Exercise	Stabilisation	Stabilisation exercise	12	35.8	
		Motor control exercise	Various	Exercises training to modify pain provocative postures and movement patterns	12	36.1	
Salavati et al. 2015 [64]	*J Bodyw Mov Ther*	Exercise	Stabilisation	Routine physiotherapy plus supervised, intensive stabilizing exercises	20	32.6	0
		Control	Various	Interferential therapy	20	29.9	0
Masharawi et al. 2013 [63]	*J Back Musculoskelet Rehabil*	Exercise	Various	Exercises	20	52.5	100
		Control		No intervention	20	53.6	100
Natour et al. 2014 [179]	*Clin Rehabil*	Pilates	Various	Pilates	30	47.8	80
		Control		No intervention	30	48.1	77
Murtezani et al. 2011 [87]	*Eur J Phys Rehabil Med*	Exercise	Individualised	High-intensity aerobic exercise	50	51.4	48
		Control	Various	IFC, TENS, ultrasound, heat	51		49
Kogure et al. 2015 [114]	*PLoS One*	Spinal manipulation	Various	Arthrokinematic Approach-Hakata method	90	60.0	60
		Sham	Various	Sham manipulation	89	59.6	64
Ozsoy et al. 2019 [92]	*Dove Med Press*	Exercise	Core Stabilisation	Core stability training	21	68.1	29
		Exercise	Various	Core stability exercise and myofascial release technique	21	68.0	31
Jousset et al. 2004 [186]	*Spine*	Multidisciplinary	Various	Multimodal rehab	43	41.4	30
		Exercise	Individualised	Active exercises	41	39.4	37
Gracia et al. 2013 [136]	*Phys Ther*	Back school	Various	Back school	74	54.2	69
		Mckenzie	Individualised	Mckenzie	74	53.7	78
Roche-Leboucher et al. 2011 [188]	*Spine*	Exercise	Various	Isotonic exercises	68	40.8	32
		Exercise	Various	Isotonic exercises	64	38.7	38
Khalil et al. 1992 [111]	*Spine*	Spinal manipulation	Stretching	Spinal manipulation	14	41.1	43
		Control		Multimodal rehab	14	48.5	50
Mannion et al. 1999 [62]	*Spine*	Exercise	Various	Isometric exercises	46	46.3	61
		Aerobics	Low-impact	Stretching and aerobic and muscle-toning exercises	47	45.2	54
		Physical agents	Various	Variuous physical agents	44	43.7	55
Mannion et al. 2001 [73]	*Spine*	Exercise	Various	Strengthening, coordination and aerobic exercises	44	46.3	61
		Aerobics	Low-impact	Stretching and aerobic and muscle-toning exercises	43	45.2	54
		Physical agents	Various	Physical therapy	40	43.7	55
Yoon et al. 2012 [125]	*Ann Rehabil Med*	Massage	Symptom guided	Deep cross-friction massge	12	50.3	58
		TENS	Various	TENS	10	53.3	60
Yang et al. 2019 [54]	*J Healthc Eng*	Exercise	Symptom guided	Exercises	5	35.0	20
		Control	Various	Manual therapy	3	50.3	100
Hicks et al. 2016 [145]	*Clin J Pain*	Control	Various	Moist heat treatment and US	31	69.5	52
		Exercise	Stabilisation	Trunk muscle training program augmented with neuromuscular electrical stimulation	26	70.7	58
Yalfani et al. 2020 [181]	*J Bodyw Mov Ther*	Water pilates	Various	Water pilates	12	25.2	100
		Pilates	Various	Pilates	12	24.7	100
Trapp et al. 2015 [56]	*J Back Musculoskelet Rehabil*	Exercise	Feedback	Exercises with biofeedback	15	45.5	33
		Control	Various	Exercises and walking	15	40.6	40
Kofotolis et al. 2016 [178]	*J Back Musculoskelet Rehabil*	Pilates	Various	Pilates	37	42.7	100
		Control		No intervention	28	41.2	100
		Exercise	Strengthening	Trunk strengthening exercises	36	39.1	100
Kuvacic et al. 2018 [170]	*Complement Ther Clin Pract*	Control		Education	15	33.6	53
		Yoga	Various	Yoga	15	34.7	40
Hernandez-Reif et al. 2001 [126]	*Intern J Neuroscience*	Massage	Various	Manual therapy	24	43.8	58
		Control	Various	Muscle relaxation exercise		36.7	50
Lewis et al. 2005 [61]	*Spine*	Exercise	Various	Active aerobic exercises	33	46.1	65
		Exercise	Individualised	Manual therapy	29	45.7	65
O’Keeffe et al. 2020 [156]	*J Sports Med*	Cognitive functional therapy	Individualised	Cognitive functional therapy	106	47.0	77
		Exercise	Various	Exercises and relaxation and pain education	100	50.6	70
Kaeding et al. 2017 [146]	*Scand J Med Sci Sports*	Exercise	Vibration	Whole body vibration	21	46.4	67
		Control		No intervention	20	44.6	70
Petrozzi et al. 2019 [160]	*Chiropr Man Therap*	App	Various	Cognitive-behavioural and exercises	54	50.1	54
		Control	Various	Manual therapy	54	50.6	59
Winter et al. 2015 [53]	*J Back Musculoskelet Rehabil*	Exercise	Rotation	Hip muscles rotation-stretching	10	45.9	45
		Exercise	Stretching	Multi-directional hip stretching	10	48.9	
		Exercise	Strengthening	Strengthening exercises	10	38.3	
Massé-Alarie et al. 2017 [152]	*Clin Neurophysiol*	Physical agents	Contraction	Magnetic neurostimulation at lumbar level	11	33.2	45
		Sham	None	Sham magnetic stimulation	10	42.1	50
Martí-Salvador et al. 2018 [122]	*Arch Phys Med Rehabil*	Spinal manipulation	Various	Manipulation	33	43.4	52
		Sham	Various	Sham spinal manipulation	33	41.7	61
Aguilar-Ferrándiz et al. 2022 [143]	*Nature*	Kinesio taping	Without tension	KT application at paravertebral musculature	29	44.0	59
		TENS		TENS	29	46.0	72
Elgendy et al. 2022 [151]	*Ortop Traumatol Rehabil*	Physical agents	Various	Shock waves	15	32.7	
		Control	Stretching, strengthening	Stretching and strengthening exercises	15	33.3	
Fukuda et al. 2021 [89]	*Braz J Phys Ther*	Spinal manipulation	Joint mobilisation	Manual therapy and lumbar stabilisation	35	35.2	53
		Spinal manipulation	Joint mobilisation, strengthening	Manual therapy and lumbar stabilisation	35	40.2	
Ma et al. 2021 [176]	*Ann Palliat Med*	Physical agents	Needling	Fu’s subcutaneus needle	30	47.7	50
		Massage	Swedish massage	Swedish massage	30	49.2	63
Maggi et al. 2022 [48]	*Aging Clin Exp Res*	Kinesio taping	No tension	Kinesio taping application at lumbar spine	57	66.8	72
		Control		Back school	62	67.8	82
Jalalvandi et al. 2022 [139]	*BMC Musculoskelet Disord*	Exercise	Stretching, strengthening	Exercises for strengthening and stretching of the back and pelvis muscles	22	37.9	73
		TENS		TENS	22	36.1	64
Atilgan et al. 2021 [93]	*J Back Musculoskelet Rehabil*	Exercise	Breathing, stabilisation	Breathing exercises and core stabilisation exercises	23	32.1	100
		Control	Stabilisation	Lumbar stabilisation	20	37.7	100
Pivovarsky et al. 2021 [140]	*Einstein (Sao Paulo)*	Sham		Sham TENS	35	40.8	69
		TENS		TENS	35	44.0	66
		TENS		TENS	35	42.6	77
Van Dillen et al. 2020 [65]	*JAMA Neurol*	Exercise	Various	Active exercises training for lumbar spine	74	42.4	68
		Exercise	Stretching, strengthening	Strength and flexibility exercises	75	42.6	55
Vibe Fersum et al. 2013 [159]	*Eur J Pain*	Spinal manipulation	Joint mobilisation	Joint mobilisation or manipulation	43	42.9	49
		Cognitive functional therapy	Unknown	Cognitive-functional therapy	51	41.0	53
Ghroubi et al. 2007 [202]	*Ann Readapt Med Phys*	Spinal manipulation	Symptom guided	Spinal manipulation	32	39.1	84
		Sham		Sham spinal manipulation	32	37.4	75
Huber et al. 2019 [162]	*BMC Musculoskelet Disord*	Walking	Walking	Guided hiking in mountains	27	52.9	52
		Walking	Walking, heat	Balneotherapy	26	53.4	54
		Control		No intervention	27	43.8	63
Werners et al. 1999 [100]	*Spine*	TENS		TENS	74	38.3	43
		Massage	Traction	Lumbar traction	73	39.2	49
Kankaanpää et al. 1999 [69]	*Spine*	Exercise	Various	Training of trunk muscles	30	39.8	37
		Control	Various	Manual therapy	24	39.3	33
Marshall et al. 2008 [82]	*Spine*	Spinal manipulation	High velocity, low amplitude, various	Isometric then concentric–eccentric exercises	12	34.3	50
		Spinal manipulation	High velocity, low amplitude	Manipulation	13	35.8	54
		Spinal manipulation	Non thrust, various	Abdominal stabilisation	12	33.9	50
		Spinal manipulation	Non thrust	Education on how to stay active	13	41.7	42
Branchini et al. 2015 [115]	*F1000research*	Spinal manipulation	Pressure	Manual therapy and fascial manipulation	11	48.0	64
		Control	Individualised	Respiratory reeducation, propioception and stretching and core stability exercises,	13	44.0	69
Batıbay et al. 2020 [177]	*J Orthop Sci*	Pilates	Various	Pilates	28	49.3	100
		Exercise		Pelvic tilt, stretching and strengthening exercises	25	48.4	100
Elabd et al. 2020 [74]	*J Appl Biomech*	Exercise	Stabilisation, stretching	Lumbar stabilisation	25	26.8	52
		Exercise	Stabilisation	Lumbar stabilisation	25	27.4	
Dadarkhah et al. 2020 [55]	*J Natl Med Assoc*	Exercise	Core stabilisation	Flexibility and strengthening and cool-down exercises	28	49.0	57
		Exercise	Core stabilisation	Flexibility and strengthening and cool-down exercises	28	50.0	57
Nardin et al. 2022 [153]	*Lasers Med Sci*	Exercise	Aerobics	Deep water running	20	42.2	80
		Sham	Aerobics	Sham	20	42.8	75
		Physical agents		Laser-therapy (THOR DD2)	20	43.1	80

US: ultrasound; TENS: transcutaneous electrical nerve stimulation; DTM: deep tissue massage; NSAID: nonsteroidal anti-inflammatory drug; PT: physical agent

## Discussion

Physiotherapy (PT) offers a comprehensive approach to managing cLBP [10]. By employing manual therapy, exercises and education techniques, PT can help reduce pain, improve mobility and enhance overall function [20]. Additionally, PT can address underlying contributing factors such as muscle imbalances, poor posture and biomechanical issues, providing long-term relief and preventing future episodes of cLBP [21]. PT helps restore physiological movement patterns and function of the lower back [22]. This allows patients to resume daily activities more efficiently and reduces limitations caused by pain [23, 24]. Thus, PT can function as a term that encompasses several approaches [25, 26].

### Patient education techniques

Patient education is a cornerstone of PT for cLBP [27]. This encompasses various approaches, including traditional ‘back school’ programs, active self-management strategies and contemporary techniques [28–30]. A primary goal of education techniques in cLBP management is to ‘demedicalize’ the patient’s condition, fostering a biopsychosocial self-management approach [31, 32]. This involves shifting attitudes away from viewing pain as solely a medical problem and towards understanding its complex interplay with psychological, social and biological factors [33]. However, attending exercise classes in a clinical setting with a physiotherapist may inadvertently reinforce patients’ perceptions of themselves as medical patients in need of treatment [33]. This could undermine demedicalization, perpetuating the belief that something is fundamentally wrong with the patient’s physical structure or health [33]. The existing literature presents a mixed picture regarding the outcomes of combining education and exercise to manage cLBP. While some studies have reported positive effects [34–38], others have yielded less favourable results [39–42].

The so-called ‘back schools’ are educational programs designed to prevent and rehabilitate back pain [43]. The original back school comes from Sweden and aims to raise patients’ awareness of their clinical condition while improving mobility, flexibility and strength through specific exercises [44, 45]. Historically, back school programs in hospital settings often follow a top-down approach, linearly presenting information and focusing on basic illness information [46]. However, contemporary research generally supports the effectiveness of back-school programs in managing cLBP [43, 47–49]. In the articles analysed in the present investigation, we found an anecdotal comparison suggesting that hirudotherapy may be more effective than back school in managing certain cases of cLBP [50]. The available data are limited and lack the rigour of controlled clinical trials.

In the last decades, a new biopsychosocial back school approach, based on the Health Action Process Approach, has been shown to be more effective in short- and long-term outcomes than the traditional back school [51]. This interdisciplinary, patient-oriented program is delivered through interactive sessions in which the trainers actively involve patients in the educational process, using a combination of methods (short lectures, group discussions, small-group work, practice and individual work) [51]. Educational processes comprehended basic knowledge about back pain (e.g. epidemiology, risk factors, development, disorders, therapy) regarding physical, psychological (fear-avoidance-endurance model, coping behaviour) and social aspects [51].

Another promising educational approach to managing cLBP involves the Movement System Impairment (MSI) classification model [52]. MSI utilises a standardised examination to identify specific movement and postural impairments associated with cLBP symptoms [52]. Treatment based on the MSI model focuses on educating patients and prescribing exercises designed to correct the identified impairments [52]. The primary goal is to minimise excessive lumbar spine movements, encourage movement in other joints, and avoid extreme spinal postures [52]. While the MSI-based approach appears logical, comparative studies have not demonstrated significant differences in outcomes compared with symptom-guided stretching and strengthening exercises in individuals with cLBP [52].

Physiotherapists also teach exercises that patients can perform at home to maintain their progress and manage future flare-ups; moreover, periodic remote monitoring or reminders of having to perform the prescribed exercises appear to improve outcomes [53, 54]. The literature provides evidence of the non-inferiority of home-based exercises when guided by qualified personnel [55]. Sensory feedback during motor re-education could also be considered a different form of education, which could be applied as a supplement [56]. Evidence exists for the beneficial effects on self-reported pain and sensory perception, suggesting that simple feedback interventions could significantly improve outcomes of standard treatment [56].

### Motor re-educational program and therapeutic exercise

A wide range of re-educational techniques fall under the category “therapeutic exercise” (TE), which can be defined as "*movement prescribed to correct impairments, restore muscular and skeletal function and maintain a state of wellbeing*" [57]. TE is a core component of a motor re-educational program, which can also involve Adapted Physical Exercise (APE) [57, 58]. APE is carried out on people with disability in a phase of clinical stability to ease their return to movement [58]. Under the guidance and following the prescription of a physiatrist, physiotherapists administer personalised TE programs that often incorporate components such as core strengthening, flexibility improvement, spinal stabilisation, and overall fitness training [59–65]. These programs are tailored to meet the specific needs of each patient and address the underlying causes of their LBP [59–65]. Results of TE usually include increased muscle strength, improved joint range of motion (ROM), reduced pain and an overall improvement in QoL [66, 67]. TE can be “active” (if the patient himself performs the movement) or “passive” (if the movement of the body segment is entirely in the hands of the physiotherapist) [62, 68]. If patients contribute to the movement while the physiotherapist provides assistance, it is referred to as an active-assisted technique [62]. Nowadays, passive mobilisation can also be applied through several devices, even remotely at home, but always under the supervision of a physiatrist or physiotherapist and with guidance from technological aids [68]. The physiatrist will consider the patient’s clinical and functional status when selecting the most appropriate treatment approach. Active techniques are generally preferred whenever possible, as they empower patients to take an active role in their recovery and can lead to more sustainable long-term outcomes [69]. Active patient engagement enhances treatment adherence and improves long-term outcomes significantly [70], and individualised approaches seem to be even more effective than standardised ones [71]. In any case, it has not yet been established which type of mobilisation is more appropriate for managing LBP, and there is evidence in the literature of therapeutic failures (e.g. lumbar rotations) to date [72]. TE can improve blood flow and reduce inflammation, promoting healing, reducing muscle tension, improving circulation and alleviating pain [57]. Introducing low-impact aerobic exercise programmes could further improve results [73]. Sometimes the exercises are not focused exclusively at the lumbar level but consider the entire spine, especially in cases of more widespread alterations (e.g. anteposed neck) [74], or consider pelvic floor muscles training to coactivate superficial and deep core muscles, resulting in significantly better improvements in pain and disability scores compared with routine treatment alone [75].

TE has demonstrated comparable efficacy to pharmacotherapy in managing cLBP [76]. Additionally, TE offers the potential advantage of improving QoL by reducing the risk of adverse side effects often associated with medications [76]. Lastly, modern technology has facilitated the remote delivery of TE through telerehabilitation [77]. Current research suggests that various methods, such as smartphone applications, can effectively manage cLBP using this approach [78]. Telerehabilitation offers several advantages, including increased patient convenience (eliminating the need for in-clinic visits), reduced healthcare costs and potentially lower environmental impact [78–83]. However, potential drawbacks include less physiatrist oversight, which could compromise treatment efficacy [78–83]. Studies evaluated in the present study suggest that supervised TE interventions typically demonstrate superior outcomes compared with unsupervised approaches [78–83].

Both exercises with moderate and high loads seem to positively stimulate the muscular and articular apparatus, improving strength, mobility and pain [84, 85]. Patients who undergo low-load exercises seem to obtain better results; this could be explained by the fact that they can focus more on performing correctly and specific exercises to their condition rather than lifting higher loads [84, 85]. Conversely, the higher load results in excellent strength improvement related to greater muscle stimulation [84, 85].

Comparative studies have also investigated the efficacy of high-intensity strengthening (HIT) versus moderate-intensity strengthening (MIT) for managing cLBP [86, 87]. HIT significantly improved disability scores and exercise capacity during and after a 12-week supervised exercise therapy program [86]. High-intensity aerobic exercise has also shown positive effects for patients with cLBP [87].

Another possible approach is lumbar stabilisation [88, 89]. Lumbar stabilisation exercises (e.g. hamstring stretch, pelvic tilt, bridge, side plank on knees) are specifically designed to strengthen the trunk and core muscles and thus support the lower back [88, 89]. These exercises aim to decrease joint instability, which can lead to pain, spinal cord impairments or damage to nerve roots [88, 89]. The benefits of lumbar stabilisation exercises include reduced LBP, improved posture, increased neuromuscular control and increased strength and endurance of spinal muscles [88, 89], even in people with sedentary occupations [90]. Physiotherapists could make use of closed or open kinetic chain exercises [91]. An open kinetic chain exercise isolates a limb’s movement, such as leg extensions on a machine [91]. On the other hand, closed-chain exercises involve the patient’s foot or hand being fixed against something, mimicking real-world activities such as squats [91]. Physiotherapists use both types to target specific muscles or improve functional movement for different goals [91]. Literature reports a possible superiority of lumbar stabilisation in the closed chain modality [91]. Stronger core muscles support the spine, while better flexibility allows for pain-free movement [34]; core stabilisation could also be combined with other types of motor re-education (e.g. myofascial techniques) [92], and the addition of breathing exercises has been suggested as a tool to improve outcomes (mostly pain, fatigue and sleep quality) [93]. A lumbar stabilisation approach is assumed to offer greater benefits than dynamic strengthening and Pilates [94]. A possible reason for this is that stabilisation exercises incorporate a drawing-in technique highly effective in simultaneously activating the transversus abdominis and multifidus muscles, fostering a pattern that primes the deep abdominal and multifidus muscles in a feedforward manner, which aids in preserving their supportive function and ensures they work in harmony with the overarching muscle groups [94]. During stabilisation exercises, deep focus is also given to the specific postural abnormalities shown by patients, making it possible to individualise treatment [95, 96]. Moreover, lumbar stabilisation exercises enhance the lumbar extensors, improve functional ability and increase the lumbar range of motion [94–96]. Impaired function or weakness of the lumbar erector spinae, which provides lumbar stability during sustained posture and movements, may result in cLBP [97]. Its activity can be successfully promoted by proprioceptive neuromuscular facilitation (PNF) training, focused on alternated isometric contractions of the trunk flexor and extensor muscles against maximum resistance, alternated concentric and eccentric contractions of trunk agonists, and on trunk movement in rotational and diagonal directions using bilateral diagonal limb movements [97]. PNF training, providing lumbar stability in both static and dynamic conditions, seems to be effective in reducing pain intensity and functional disability and improving patient satisfaction [97]. When combined with walking, lumbar stabilisation exercises effectively reduce pain and improve muscle endurance in patients with LBP [98]. This combination may also help prevent recurrences [98]. Lumbar traction is another technique widely used in the past for LBP but increasingly less today [99, 100]. Lumbar traction stretches the lower back to ease lower back pain [99, 100]. It can be done with a machine or directly by a physical therapist [99, 100]. While effective for some patients in reducing pain and improving mobility, research is not conclusive, and side effects can occur (e.g. muscle soreness, pain, headache) [99, 100].

Emerging evidence suggests that TE targeting the lower limbs may be valuable to manage cLBP in recreational runners [101]. Current evidence has shown that individuals with cLBP often exhibit impairments in their lower limb muscles, including weakness in knee extensors and reduced hip extensor activation and endurance [101]. These impairments can compromise shock absorption during activities such as running, potentially transferring excessive forces to the lower back and contributing to pain [101]. Strengthening lower limb muscles may help alleviate pain and improve function by reducing loading on the lower back [101]. Therefore, lower limbs TE warrants consideration as an alternative or complementary approach for treating cLBP [101]. This strategy holds promise for superior re-educational outcomes, particularly improving running ability, knee extension strength and overall running gait [101]. However, it is important to acknowledge that the current research primarily focuses on the effects on runners, and further investigation is needed to determine the generalizability of these findings to other populations with cLBP [101].

Motor control exercises (MCE) are another category widely applied to manage cLBP [102]. Spine control and stability are usually abnormal in people with cLBP (e.g. delayed activation of the deep trunk muscles in dynamic tasks, fat infiltration in the paravertebral muscles and increased stiffness to compensate for low mobility) [103]. MCE seems to help reduce these burdens through a motor-learning approach to improve spine and pelvis control, posture, movement and muscle coordination with re-educational exercises [103, 104]. Compared with sham therapy (i.e. detuned ultrasound therapy and detuned short-wave therapy), MCE (i.e. re-education of the multifidus and transversus abdominis muscles, pelvic-floor muscles, breathing control and control of spinal posture and movement) improve activity and global feeling of recovery but not pain at 2 months, with effects lasting up to 1 year [102, 105]. When the effects of a program based on individualised MCE combined with cognitive-behavioural therapy (CBT) are compared with those of a standard motor program alone (i.e. passive spinal mobilisation, stretching, muscle strengthening and postural control), MCE plus CBT produced greater benefits on disability, kinesiophobia, catastrophising and QoL; probably these patients had better coordination of spinal muscles with less lumbar dysfunction and need of compensatory strategies achieving better functional outcomes [106, 107]. Adding CBT can help improve therapy outcomes for patients’ awareness of the problem and help them adopt a more functional compensatory strategy [106, 107]. When MCEs are compared with lumbar stabilisation exercises, no differences in pain or disability are found, and both approaches appear effective [108].

Graded activity is a form of TE that progressively increases demands on the patient, with incremental goals that lead to greater activity tolerance [109]. It usually works best in deconditioned patients [109]. Compared with MCE, graded activity seems superior to MCE in patients with low spine instability, low coping, low fear and low psychosocial risk [110]. Conversely, MCE have greater benefits in patients with self-reported signs of clinical instability [110]. This is consistent with the nature of the two approaches (one focused on gradually improving patients’ performances, the other on spine instability) and their clinical rationale [109, 110].

Stretching is another component of a motor re-educational program that could benefit the management of cLBP [111]. By improving flexibility, reducing muscle tension and increasing ROM, stretching can help alleviate pain and discomfort. Systematic stretching manoeuvres enhance the functional gains of patients with cLBP [111]. Isostretching is a form of static stretching where a muscle is stretched to its maximum point and held in that position for an extended period. Unlike dynamic stretching, which involves movement, isostretching is a static exercise [112]. Tension is produced within the muscle and its connecting tissues when a muscle is stretched [112]. By holding this stretch, the muscle fibres gradually lengthen, achieving increased flexibility and ROM [112]. Isostretching is a valuable technique to reduce pain and improve function, patient satisfaction and QoL in patients with cLBP [112].

### Manual therapy/manipulation

Another possible PT approach to cLBP is Manual Therapy (MT) (or spinal manipulation): spinal manipulation differs from mobilisation as it involves high-velocity thrusts that exceed the joint’s normal range of motion, often producing audible clicks [113]. MT can reduce pain if applied by specific methods, such as the Arthrokinematic Approach-Hakata method [114] or fascial manipulation [115]. When followed by targeted active exercises, it significantly decreases functional disability and further attenuates pain intensity [116]. Electromyographic (EMG) analysis has shown that MT can effectively reduce abnormal electrical signals and activity within muscles [117]. Some authors have reported superior short- and long-term outcomes of spinal manipulation compared with back school or individual physiotherapy regarding functional improvement and pain relief [48, 118, 119]. However, while confirming the effectiveness of spinal manipulation, different techniques and approaches do not show significant differences [120–122]. Moreover, comparative studies on region-specific versus non-region-specific spinal manipulation have demonstrated no immediate differences in pain intensity or pressure pain threshold [29]. Some studies have reported a more pronounced effect of MT on pain reduction compared with functional improvement [123]. This disparity may be attributed to the limitations of MT in addressing psychosocial factors or other underlying contributors to disability [123]. Further, more rigorous research is necessary to address the observed heterogeneity in study outcomes and establish more definitive conclusions regarding the effectiveness of various interventions.

### Massage techniques

Massage techniques (e.g. lumbar tender point deep massage and deep cross-friction massage) have emerged as valuable adjuncts in managing various musculoskeletal conditions, including cLBP [124–126]. Deep tissue massage is designed to modify soft tissue structures and restore muscle mobility [124]. The physiotherapist gradually applies pressure to the tissues until they respond with relaxation [124]. Optimal therapeutic effects are achieved by positioning the patient’s muscles in an extended position [125]. The massage therapist focuses on the muscle belly and tendon-bone attachments, aiming to soften the tendon and influence muscle stretch receptors [126]. The ultimate goal is to release chronic muscle tension and restore physiological movement patterns [127], reducing pain and disability at the end of the treatment and in the medium term, compared with transcutaneous electrical nerve stimulation (TENS) [125]. Massage can also be applied following specific methods, such as the Gua Sha Therapy, an instrument-assisted “pressure-stroking” of a lubricated area of the body surface [128], or Thai traditional massage, a form of bodywork that combines acupressure, stretching and passive yoga to improve circulation, flexibility and overall wellbeing by applying pressure to specific points on the body using hands, feet, elbows and knees [129].

### Postural therapies

Postural therapies (e.g. McKenzie Method, Souchard, and Feldenkrais) emphasise improving body alignment and reducing spinal strain to alleviate cLBP [79, 130, 131]. These approaches incorporate exercises, stretches, and educational components to address postural imbalances and enhance spinal alignment. These therapies can relieve pain, improve mobility, and prevent future flare-ups by reducing strain on the spine. Moreover, these methods empower patients by teaching them self-care strategies and proper body mechanics, enabling them to manage their cLBP actively.

The Feldenkrais method is a postural therapy that uses gentle movement and directed attention to help people learn new and more effective moving methods [79]. The process is based on the concept that our movement patterns are internalised, and, by becoming more aware of our habits, we can improve them for the better [79]. Feldenkrais lessons typically involve lying on a mat and slowly moving through small, precise movements [79]. At the same time, an operator guides participants verbally, helping them focus on their sensations and how their bodies are moving in space [79]. The Feldenkrais method effectively improves body awareness and posture, increases flexibility and ROM and reduces pain, stress and anxiety [79].

McKenzie’s method, also called Mechanical Diagnosis and Therapy (MDT), for LBP is based on specific exercises, repetition and an educational approach [132]. MDT empowers patients to learn exercises and techniques to manage their pain, reducing reliance on healthcare professionals for long-term care [132]. Unlike some passive treatments, the McKenzie Method uses specific repeated movements to improve mobility and function in the spine [132]. Patients undergoing MDT are classified into three categories (i.e. derangement, dysfunction and postural) according to their response to repeated movements and maintaining precise positions for long periods [131]. This is used to apply a tailored re-educational program and enable patients to self-manage LBP’s symptoms [131]. The validity of the McKenzie method has been demonstrated, with improvement in pain measures compared with placebo [131]. MDT actively involves patients through education for back, neck and extremity disorders, encouraging patients to learn the exercises and techniques to manage their pain [133]. Comparative studies have shown that MCE and MDT may yield similar short- and long-term outcomes regarding muscle recruitment, pain and function [134, 135]. However, the duration of treatment appears to be a critical factor [134, 135]. A limited number of sessions, such as 12 sessions over eight weeks, might not be sufficient to achieve optimal results [134, 135]. MDT has also been compared with back school [136]. Both MDT and back school seem effective in reducing cLBP-related pain and disability, with the latter having slightly better results [136]. MDT reduces work-related LBP more than interferential current, ultrasound and heat [137].

Souchard is a postural therapy that addresses underlying postural imbalances to reduce cLBP symptoms and improve overall spinal health [130]. The method involves a series of exercises and stretches designed to improve flexibility and strengthen core muscles [130]. Souchard can also help patients develop better body awareness and learn how to maintain good posture throughout the day [130]. This technique effectively improves pain, function and QoL aspects (emotional, limitations in physical functioning, vitality and mental health) in cLBP [130].

### Physical agents

PT can be combined with physical agents such as heat, cold, electricity, vibration, ultrasound and electromagnetic waves to reduce pain and inflammation, promote tissue healing, improve muscle and joint function and re-educate movement. The main categories include thermotherapy (heat), cryotherapy (cold), electrotherapy (electric current), magnetotherapy (magnetic fields) and laser therapy (laser light). Antalgic electrotherapy (i.e. TENS, interferential current) is particularly useful for pain reduction and is widely applied in LBP; it is essential to acknowledge that the existing literature presents conflicting evidence regarding the efficacy of antalgic electrotherapy intervention on function and mobility compared with traditional approaches like mobilisation and exercise [138–140]. While some studies report limited benefits [138], others suggest positive outcomes [139]. The more remarkable agreement is present, however, regarding the reduction of pain [139], both if the TENS was applied in conventional (continuous stimulation at 100 Hz for 100 µs with sensory intensity) and burst mode (stimulation at 100 Hz modulated at 2 Hz for 100 µs with motor-level intensity) [140]. On the other hand, the association of exercises (e.g. core strengthening, aerobic exercises) and TENS seem to be a valid option, adding up the benefits of the two techniques [141, 142]. This association produces greater improvements in pain, disability, anxiety, depression and sleep patterns than those obtained by kinesio taping and exercises [143]. In the literature, data about how effectiveness is affected by the type of applications, site of application, treatment duration, optimal frequencies and intensities are broadly lacking [144]. Electrotherapy could also be used for muscle stimulation and strengthening: neuromuscular electrical stimulation, electrical impulses to stimulate nerves and cause muscles to contract, and it has shown necessary improvements in both performance-based and self-reported measures of function [145]. Whole body vibration (WBV) is a viable and effective instrumental physiotherapy approach which uses oscillatory muscle stimulation: feet are placed on a vibrating platform [146]. Vibrations spread to the whole body, stimulating muscle activation through tonic reflexes [147]. Mechanical vibration could strengthen muscles, particularly paravertebral and lower limbs, and improve balance and neuromuscular performance, thus reducing pain and improving mobility [146–148]. To achieve the same objectives, vibration can also be applied to the cables of a sling suspension system while patients perform sling exercises (Neurac system) [149]. The combination of vibratory stimulation and sling exercise allows for high-level stimulation of the neuromuscular system, which is particularly effective for lumbar stabilisation [149]. However, a multidisciplinary approach incorporating physical therapy could be better at optimising long-term pain reduction and functional outcomes in patients with LBP [150]. Shockwave therapy is a non-invasive treatment for cLBP that involves delivering focused acoustic waves to the affected area [151]. This therapy can reduce pain, improve function, reduce medication reliance and minimise side effects, mainly if administered alongside a standard exercise program [151].

A combination of repetitive peripheral magnetic stimulation (RPMS) and motor training of the superficial multifidus muscle might favour motor learning and brain plasticity more than training alone [152]. Brain plasticity improves the corticomotor control of the multifidus and lumbopelvic spine during activities of daily living without generating pain [152].

Photobiomodulation therapy, or low-level laser therapy, is a non-invasive treatment that uses light energy to stimulate cellular processes and promote healing [153]. When applied to cLBP, photobiomodulation can offer several benefits [153]. It can help alleviate pain, reduce muscle tension, and accelerate tissue repair by reducing inflammation, improving blood circulation and modulating pain signals [153]. Additionally, photobiomodulation may enhance the effectiveness of other treatments, such as physical therapy or deep water running, leading to improved outcomes for individuals with LBP [153].

Electro-acupuncture uses electrical current to stimulate acupuncture needles [154]. This technique is believed to enhance the therapeutic effects of traditional acupuncture [154]. While electro-acupuncture can effectively manage cLBP, the exact mechanisms by which it works are not fully understood [154]. Electro-acupuncture may help to reduce pain, improve muscle function and promote healing by stimulating the release of endorphins and other neurotransmitters [154]. Emerging evidence suggests that combining electro-acupuncture with back exercises may effectively manage cLBP [154]. This synergistic combination could enhance pain relief and functional outcomes [154].

In addition to physical therapy, the back care pillow helps decrease pain intensity and disability and improve the ROM [155]. Its use has multiple benefits, such as maintaining the appropriate lumbar curvature and decreasing back muscle spasms and intra-disc pressure by changing the distribution of loads between discs [155].

### Other miscellaneous approaches

A variety of other approaches have been employed in the management of cLBP. The efficacy of these regimens suggests that TE alone cannot fully account for the management of cLBP. Psychological factors, such as altered attentional focus and reduced pain-related avoidance behaviours, are crucial in mitigating the effects of deconditioning [156]. Cognitive-behavioural therapies (CBT) are effective in addressing these psychological components [156–159]. CBT (involving education with making sense of pain, graded movement exposure and lifestyle changes) was more effective than manual therapy and exercise (MT-EX) in reducing disability at 6-months and 1-year follow-up [156], but also at 3-year follow-up without concomitant reductions in pain intensity, suggesting a decoupling of the pain – disability relationship, probably related to the reductions in anxiety and depression and pain related fear regarding work [157], in contrast to previous studies with positive results both in disability, fear-avoidance beliefs and pain [158, 159]. Instead, no additional benefits were found with the use of an internet-delivered CBT program (MoodGYM) to physical treatments in those patients with non-specific cLBP at medium risk of ongoing disability [160].

Another alternative technique is therapeutic climbing, which offers a unique approach that combines physical and psychological benefits [161]. It provides a structured environment for core and trunk muscle strengthening while simultaneously addressing pain-related fear and avoidance behaviours [161]. Therapeutic climbing may facilitate a more positive body image and improved coping mechanisms by shifting attention away from pain and towards physical accomplishment [161]. Guided mountain hiking is another possible alternative to benefit individuals with cLBP [162]. The gradual inclines in guided hikes allow for a controlled increase in physical demands, reducing the risk of strain [162]. Hiking strengthens muscles in the legs, core and back while improving flexibility and range of motion [162]. As a weight-bearing activity, hiking helps maintain bone density and enhance balance [162]. Being in nature can provide a distraction from pain and reduce stress, both of which can contribute to cLBP [162].

Kinesio taping (KT), or K-tape, is a therapeutic technique that could aid during PT [143, 163]. KT involves applying elastic adhesive tape to the skin [164]. The tape is designed to mimic the elasticity of human skin [164]. It should provide various benefits, including pain relief, supporting muscles and joints, reducing inflammation, improving circulation and healing and enhancing proprioception (body awareness) [165]. The tape is applied in specific ways depending on the intended outcome (e.g. paravertebral application in case of LBP) [165]. KT can effectively reduce pain in LBP patients, regardless of whether it is applied with or without tension [165]. Moreover, when used with tension, KT has demonstrated the ability to improve disability, ROM and pain at short-term post-application [164] with positive effects on ROM and disability sustained for long [165]. KT might be helpful, mainly when used alongside PT [143, 163].

### Complementary medicine techniques

Various complementary medicine techniques have been applied to treat cLBP. Yoga combines physical postures, breathing exercises and meditation. It aims to promote physical and mental wellbeing [166]. Immersive yoga-based lifestyle programs can effectively manage pain-related disability and improve spinal flexibility [167]. These programs often demonstrate superior outcomes compared with traditional physical exercise regimens [166–168]. Compared with PT, yoga seems to produce similar results, with sustained improvements observed at 1 year, regardless of the maintenance strategy employed, without increasing rates of adverse events, primarily involving joint and back pain [169]. Yoga has the advantage of having demonstrated its effectiveness in addressing not only pain intensity and functional disability but also psychological factors such as depression and anxiety, which can significantly influence pain perception [170, 171]. Shiatsu is a Japanese bodywork therapy that involves applying pressure to specific points on the body [172]. Current literature reports that combining Shiatsu with standard care for cLBP could lead to improvements in pain symptoms and overall QoL shortly after the completion of Shiatsu treatments [172]. Tai Chi and Qi Gong are ancient Chinese practices that focus on cultivating vital energy, or *qi* [173, 174]. Both involve slow, fluid movements coordinated with breathing and aim to improve physical and mental health [173–175]. While research on the effectiveness of Tai Chi for managing cLBP is ongoing, existing studies offer mixed findings. Some authors suggest that Tai Chi may protect the nervous system in ageing individuals with non-specific LBP while also alleviating pain [173]. On the other hand, others report that, while Tai Chi can reduce pain, it may not improve lower limb proprioception in patients with chronic nonspecific LBP [174]. Qigong, instead, significantly reduces pain and improves range of motion and core muscle strength while ameliorating heart rate, respiratory rate and mental status [175].

Fu’s subcutaneous needling is a traditional Chinese medicine technique that involves inserting fine needles into specific points beneath the skin [176]. Unlike acupuncture, which targets more profound points, Fu’s subcutaneous needling focuses on superficial layers [176]. This technique stimulates circulation, reduces pain and improves overall health [176]. It is often used to treat conditions like chronic pain, stress and insomnia. Reports regarding the benefits of Fu’s subcutaneous needling in cLBP are available [176].

Pilates, developed in the early twentieth century, is a physical fitness system that focuses on strengthening the body, improving flexibility, and enhancing posture. Multiple studies have demonstrated the effectiveness of Pilates in managing cLBP [177–180]. Pilates improves pain, function, and QoL, including functional capacity and vitality [177–181]. A comparative study found that mat and water Pilates programs can benefit individuals with LBP [181]. However, the study identified a potential limitation of Pilates in improving balance [181]. Further research may be necessary to explore the combination of Pilates with additional exercises to address balance deficits.

### Multidisciplinary approaches

In the present review, different techniques have been discussed individually. Research increasingly supports the superiority of a multidisciplinary approach in managing cLBP [182–190]. This holistic approach encompasses physical training, occupational therapy, workplace interventions, back school, education, relaxation training, psychological support and cognitive-behavioural stress management methods [182–190]. While PT can be valuable, combining it with these additional interventions often leads to more comprehensive and long-lasting outcomes [182–190]. The effectiveness of multidisciplinary approaches can vary, and some studies have reported mixed results [191]. However, the growing body of evidence suggests that a holistic approach is generally more beneficial in addressing the complex nature of chronic pain, which often involves physical, psychological and social factors.

## Conclusions

Managing cLBP is challenging. While individual investigations highlight the efficacy of a specific intervention, most cLBP management strategies share common elements. Combining key elements from various approaches may prove crucial. Although this result is not decisive in treatment, it paves the way for further research considering a more holistic and multidisciplinary approach.

## Supplementary Information

Supplementary Material 1

## Data Availability

The datasets generated during and or analysed during the current study are available throughout the manuscript.
